# Sexual dimorphism in brain transcriptomes of Amami spiny rats (*Tokudaia osimensis*): a rodent species where males lack the Y chromosome

**DOI:** 10.1186/s12864-019-5426-6

**Published:** 2019-01-25

**Authors:** Madison T. Ortega, Nathan J. Bivens, Takamichi Jogahara, Asato Kuroiwa, Scott A. Givan, Cheryl S. Rosenfeld

**Affiliations:** 1 Bond Life Sciences Center, University of Missouri, Columbia, MO 65211 USA; 2 Biomedical Sciences, University of Missouri, Columbia, MO 65211 USA; 3 DNA Core Facility, University of Missouri, Columbia, MO 65211 USA; 4 Division of Bio-Resources, Frontier Science Research Center, University of Miyazaki, Miyazaki, 889-1692 Japan; 5 Department of Biological Sciences, Faculty of Science, Hokkaido University, Sapporo, Hokkaido 060-0810 Japan; 6 Informatics Research Core Facility, University of Missouri, Columbia, MO 65211 USA; 7 Molecular Microbiology and Immunology, University of Missouri, Columbia, MO 65211 USA; 8 MU Informatics Institute, University of Missouri, Columbia, MO 65211 USA; 9 Thompson Center for Autism and Neurobehavioral Disorders, University of Missouri, Columbia, MO 65211 USA

**Keywords:** Sexual differentiation, Organizational-Activational programming, Rodent, Endangered animals, SRY, Steroid hormones, Testosterone, Estrogen, RNAseq, Bioinformatics

## Abstract

**Background:**

Brain sexual differentiation is sculpted by precise coordination of steroid hormones during development. Programming of several brain regions in males depends upon aromatase conversion of testosterone to estrogen. However, it is not clear the direct contribution that Y chromosome associated genes, especially sex-determining region Y (*Sry*), might exert on brain sexual differentiation in therian mammals. Two species of spiny rats: Amami spiny rat (*Tokudaia osimensis*) and Tokunoshima spiny rat (*T. tokunoshimensis*) lack a Y chromosome/*Sry*, and these individuals possess an XO chromosome system in both sexes. Both *Tokudaia* species are highly endangered. To assess the neural transcriptome profile in male and female Amami spiny rats, RNA was isolated from brain samples of adult male and female spiny rats that had died accidentally and used for RNAseq analyses.

**Results:**

RNAseq analyses confirmed that several genes and individual transcripts were differentially expressed between males and females. In males, seminal vesicle secretory protein 5 (*Svs5*) and cytochrome P450 1B1 (*Cyp1b1*) genes were significantly elevated compared to females, whereas serine (or cysteine) peptidase inhibitor, clade A, member 3 N (*Serpina3n*) was upregulated in females. Many individual transcripts elevated in males included those encoding for zinc finger proteins, e.g. zinc finger protein X-linked (*Zfx*).

**Conclusions:**

This method successfully identified several genes and transcripts that showed expression differences in the brain of adult male and female Amami spiny rat. The functional significance of these findings, especially differential expression of transcripts encoding zinc finger proteins, in this unusual rodent species remains to be determined.

**Electronic supplementary material:**

The online version of this article (10.1186/s12864-019-5426-6) contains supplementary material, which is available to authorized users.

## Background

The undifferentiated gonad and brain are programmed to be either male or female in most sexually reproducing species. Brain sexual differentiation in therian mammals is generally influenced by a surge in gonadal steroid hormones during embryo development followed by a second surge later in adulthood. This paradigm is termed “organization-activational programming” and occurs in a sex-specific manner [1–3]. The organizational period is typified by spikes in androgens and/or estrogens during development and results in permanent masculinization and defeminization of the brain in males [4–7]. Masculinization of several brain regions, including the hippocampus, is modulated by aromatization of testosterone to estrogen [4–7]. Manifestation of sex-dependent behaviors, especially male-specific traits, requires a second spike in steroid hormones at adulthood (“activational period”) [8–13]. The salient question remains as to the potential contribution of sex chromosome-associated genes in guiding brain sexual differentiation in therian mammals.

Sex-determining region Y *(Sry*) resides on the Y chromosome in therian mammals, which includes placental mammals and marsupials [14–18]. This gene serves as a transcriptional activator modulating testis development, and thereby, it was coined the testis determining factor (TDF) [19–21]. SRY includes an HMG box as a DNA-binding domain [22] that activates in the testes Sry-related box 9 (*Sox9*) [23], cerebellin 4 precursor gene (*Cbln4*) [24], transcription factor 21 (*Tcf21*, former also called *Pod1*) [25], and neurotrophin 3 (*Nt3*) [26]. However, the role of *Sry* in brain sexual differentiation is uncertain.

The four core genotype (FCG) mouse model has been used in elucidating the neural effects of *Sry* relative to other Y chromosome associated genes [27–34]. In this model, *Sry* is deleted from the Y chromosome and re-inserted as a transgene on an autosomal chromosome in both XX and XY chromosome bearing mice [27]. The resulting breeding scheme gives rise to the FCG mouse model as offspring from these mated pairs can be one of four different genotypes: XX*Sry*^*−*^(karotypically and gonadally female, naturally lacking *Sry*), XX*Sry*^*+*^ (karoytypically female but gonadally male due to presence of autosomal *Sry* transgene), XY*Sry*^*−*^ (karyoptically male but gonadally female due to deletion of endogenous *Sry* gene), and XY*Sry*^*+*^ (karyotypically and gonadally male) [28]. Results to date with this model reveal that *Sry* and other sex chromosome associated genes interact with steroid hormones to affect neurobehavioral programming. Gonadectomized XX females consume more food and show increased adiposity relative to XY mice [29]. On the other hand, intact XX*Sry*^*−*^ and XY*Sry*^*−*^ mice have increased activity levels, consume less food, and show enhanced anxiety-like behaviors [30]. Social and parenting behaviors are also influenced by *Sry* and other Y chromosome associated genes [32, 35]. This model indicates *Sry* regulates neural expression of progesterone receptor (PR) in the anteroventral periventricular nucleus (AVPV), medial preoptic area (MPOA), and ventromedial nucleus, gamma-aminobutyric acid (GABA)/serotonin/dopamine-related genes within the frontal cortex, and growth hormone (*Gh*) expression in the hypothalamus [27, 33, 34].

To ascertain the potential role of the Y chromosome and *Sry* in regulating brain sexual differentiation, a better approach though would be to examine the brain transcriptome profile in males and females of therian mammals where the males lack a Y chromosome/*Sry* and both sexes possess an XO system. Such is the case with two *Tokudaia* (Amami [Ryukyu] spiny rat- *Tokudaia osimensis* and Tokunoshima spiny rat- *T. tokunoshimensis*) and two *Ellobius* (Transcaucasian mole vole- *E. lutescens* and Zaisan mole vole- *E. tancrei*) species [36–41]. Both *Tokudaia* species are critically endangered, and thus, it is vital to begin to understand how gonad and brain sexual differentiation occurs in them. In Amami spiny rats, two possible genes that might guide testes formation are chromobox protein homolog 2 *(Cbx2)*, which acts upstream of *Sry*, and *Er71*, also called Ets variant 2 (*Etv2*), which is upregulated by *Sry* in species containing this gene [42, 43]. However, it is not clear which genes or transcript isoforms show sex differences in this *Tokudaia* species. Based on the current status of Amami spiny rats, it is not permissible to obtain brain samples from embryonic or neonatal individuals. Thus, it is not possible to determine which gene(s)/transcript(s) might guide gonad and potentially brain sexual differentiation in these species. Sex differences in brain expression remain evident in adult mice, birds, and humans [44–48]. To begin to understand, neural sex differences in Amami spiny rat, the current studies sought to determine which genes/transcript isoforms show sexually dimorphic patterns of expression in the brain of this species. An ancillary goal of this study was to develop the first reference transcriptome library for Amami spiny rat that may aide in future studies designed to identify putative sex-determining gene(s)/transcript(s) in this species. RNAseq analyses was performed on whole brain samples from male and female adult individuals that died accidentally as part of a government-approved mongoose eradication project on the island in which this species inhabits. The overarching hypothesis was that there would be considerable sexually dimorphic differences in the brain transcriptomic profile with signature patterns identified in each of the sexes.

## Results

### General characterizations

**Fig. 1:**
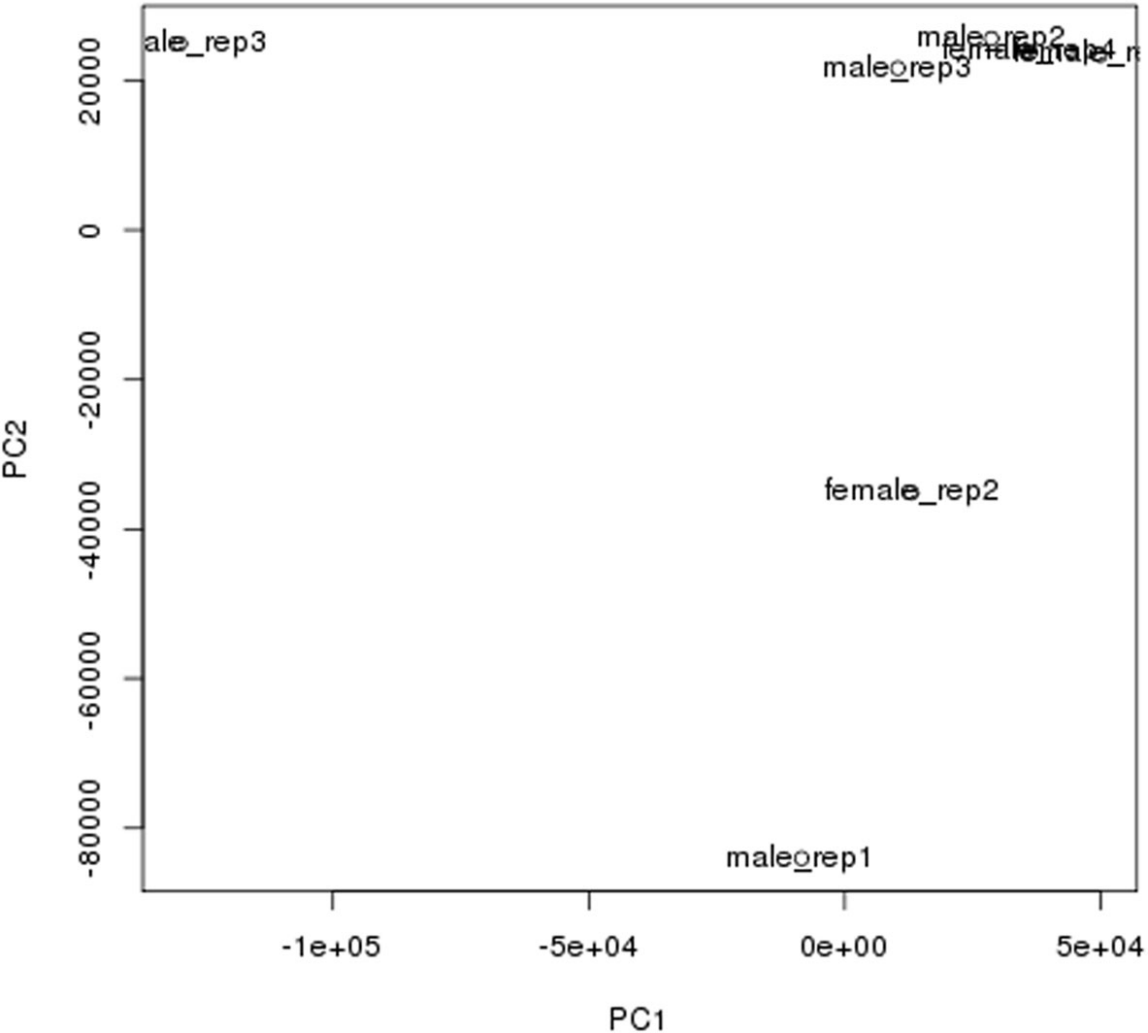
PCA analysis. Three samples were obvious outliers: male sample #1, female sample #2 and female sample #3, and thus, these samples were removed from further RNAseq expression analysis

Table 1 lists the summary statistics of the transcriptome assembly for the Amami spiny rat brain samples. A total of 569,369 transcripts were assembled with a mean length of 839.88 nucleotides (nt). 116,192 transcripts were at least 1000 nt in length. We used a BUSCO analysis to gauge quantitatively the completeness of the assembly as compared to a database of single copy vertebrate orthologs sequences [49]. The Amami spiny rat transcriptome assembly contained 76.4% of the 4104 single copy vertebrate BUSCO orthologs. 29.4% of the orthologs were also single copy in the Amami spiny rat assembly, whereas 47% were duplicated. 16.1% were fragmented and 7.5% were missing. These numbers are similar to other assembled transcriptomes for samples collected from non-inbred, wild vertebrate populations [50–53]. Full details on the transcriptome library generated for this species are included on https://www.ncbi.nlm.nih.gov/bioproject/474959. Relationships between samples were visualized by using principal component analyses (PCA). Upon plotting the PCA results, three samples were obvious outliers: male sample #1, female sample #2 and female sample #3, as evidenced by the fact these samples were at a significantly different location from other points in the PCA plot (Fig. 1). These samples were removed from further expression analysis.

**Table 1:** Transcript Assembly Metrics for the Transcriptomic Library Generated from the brain of male and female Amami spiny rats

Categories	Number of Reads or Percentage
Total Assembly Length^a^	478,202,943
Number of Assembled Transcripts^a^	569,369
Mean Transcript Length^a^	839.88
Transcript N50^a^	1751
N Transcripts > 1 k nt^a^	116,192
Longest Transcript^a^	22,701
N Transcripts with an identifiable ORF^a^	80,210
BUSCO: Complete^b^	76.4%
BUSCO: Complete Single Copy^b^	29.4%
BUSCO: Complete Duplicated^b^	47%
BUSCO: Fragmented^b^	16.1%
BUSCO: Missing^b^	7.5%
N BUSCO orthologs (vertebrate) ^b^	4104

^a^Value generated with TransRate (17)

^b^Value generated with BUSCO software (18)

### Differential expression of genes in the brain of male and female Amami spiny rats

**Fig. 2:**
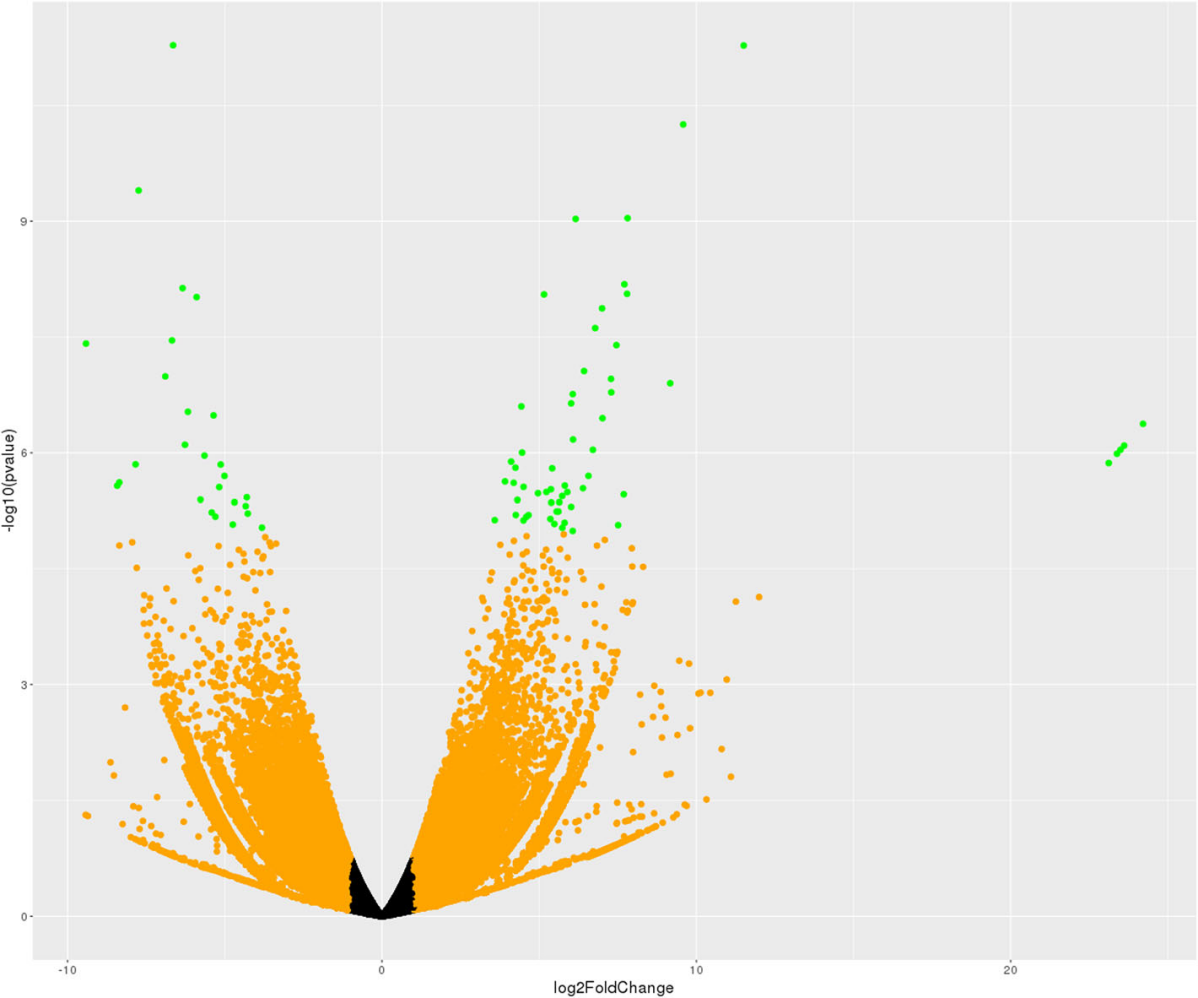
Volcano plot analyses of differentially expressed genes. The volcano plot represents the relationship of each gene’s log2 fold change vs -log10 pvalue. Orange points represent expression ratios greater than 2X (either up or down), red points represent genes whose FDR-corrected *p*-values are < 0.05 and green points are genes where both properties occur (fold-change > +/− 2X and FDR < 0.05)

The RNA-Seq data was aligned to the sequences in the transcriptome assembly, described above, using RSEM (48), which also estimates the expression levels of transcripts. We further processed gene expression levels using tximport [54]. Differential expression analysis used DESeq2 [55]. Only transcripts having a sum of counts from all samples greater than 25 were analyzed. The genes that displayed differential expression (q ≤ 0.05) in both analyses were examined further (Additional file 1). Volcano plot analyses revealed that several genes showed differential expression in the brain of male compared to female Amami spiny rats (Fig. 2). As shown in Tables 2, 34 genes were upregulated in the brain of male compared to female Amami spiny rats. These include such genes as lumican precursor (*Lum*), seminal vesicle secretory protein 5 precursor (*Svs5*), cytochrome P450 B1 (*Cyp1b1*), and a few zinc finger protein-like genes. In contrast, only four genes were downregulated in males compared to females, including serine (or cysteine) protease inhibitor, clade A, member 3 N isoform X1 (*Serpina3n*), V(D)J recombination-activating protein 2 (*Rag2*), antigen KI-67 isoform X3 (*Mki67*), and B2 bradykinin receptor isoform XI (*Bdkrb2*) (Table 3).

**Table 2:** Top genes upregulated in the brain of male compared to female Amami spiny rats

Entrez ID	Gene Symbol	Gene Name	Adjusted *P* value	Fold Change
17,022	*Lum*	ref|NP_032550.2| lumican precursor [*Mus musculus*]	9.83E-05	16.19695893
10,345	*Trdn*	ref|NP_001242951.1| triadin isoform 5 [*Homo sapiens*]	0.002669	13.1084315
17,189	*Mb*	ref|NP_001157520.1| myoglobin [Mus musculus]	0.002669	9.745618194
16,509	*Kcne1*	ref|NP_032450.1| potassium voltage-gated channel subfamily E member 1 [Mus musculus]	0.004555	12.03112079
67,473	*Slc47a1*	ref|XP_006534058.1| PREDICTED: multidrug and toxin extrusion protein 1 isoform X4 [Mus musculus]	0.004689	11.4177857
17,229	*Tpsb2*	ref|XP_006523803.1| PREDICTED: tryptase beta-2 isoform X1 [Mus musculus]	0.004689	11.59190431
11,889	*Asgr1*	ref|NP_001278061.1| asialoglycoprotein receptor 1 isoform b [Mus musculus]	0.004689	8.559833347
NA	LOC102639700	ref|XP_017170801.1| PREDICTED: zinc finger protein 709-like isoform X1 [Mus musculus]	0.005575	9.999921401
18,295	*Ogn*	ref|NP_032786.1| mimecan precursor [Mus musculus]	0.006566	10.65807665
12,491	*Cd36*	ref|XP_006535688.1| PREDICTED: platelet glycoprotein 4 isoform X1 [Mus musculus]	0.009481	8.395122091
207,151	*Slc22a19*	ref|NP_659034.1| solute carrier family 22 (organic anion transporter), member 9 [Mus musculus]	0.012094	9.473960337
66,106	*Smpx*	ref|NP_001239520.1| small muscular protein [Mus musculus]	0.012695	6.892002622
12,825	*Col3a1*	ref|NP_034060.2| collagen alpha-1(III) chain preproprotein [Mus musculus]	0.012695	9.470753107
21,743	*Inmt*	ref|NP_033375.1| indolethylamine N-methyltransferase [Mus musculus]	0.018859	8.404910004
69,563	*Mrln*	ref|NP_001291668.1| myoregulin [Mus musculus]	0.019443	7.6303154
13,179	*Dcn*	ref|NP_031859.1| decorin preproprotein [Mus musculus]	0.019443	8.25342904
17,901	*Myl1*	ref|NP_001106858.1| myosin light chain 1/3, skeletal muscle isoform isoform 3f [Mus musculus]	0.02744	6.849686453
27,047	*Omd*	ref|NP_036180.1| osteomodulin precursor [Mus musculus]	0.028951	8.601246508
1634	*Dcn*	ref|NP_598012.1| decorin isoform c precursor [Homo sapiens]	0.028951	7.901490581
20,944	*Svs5*	ref|NP_033327.1| seminal vesicle secretory protein 5 precursor [Mus musculus]	0.029739	8.768386135
17,906	*Myl2*	ref|XP_006530249.1| PREDICTED: myosin regulatory light chain 2, ventricular/cardiac muscle isoform isoform X1 [Mus musculus]	0.03194	7.130268022
208,890	*Slc26a7*	ref|NP_666059.2| anion exchange transporter [Mus musculus]	0.03194	8.375478579
NA	*H2-Ea-ps*	ref|NP_034511.2| histocompatibility 2, class II antigen E alpha precursor [Mus musculus]	0.034439	8.239364691
NA	LOC108169190	ref|XP_017177372.1| PREDICTED: probasin-like [Mus musculus]	0.035402	8.441676192
208,164	*Fam180a*	ref|NP_775551.1| protein FAM180A precursor [Mus musculus]	0.035402	7.641236964
14,181	*Fgfbp1*	ref|XP_006503775.1| PREDICTED: fibroblast growth factor-binding protein 1 isoform X1 [Mus musculus]	0.04055	7.962666492
13,078	*Cyp1b1*	ref|NP_034124.1| cytochrome P450 1B1 [Mus musculus]	0.044278	7.325271545
13,009	*Csrp3*	ref|NP_038836.1| cysteine and glycine-rich protein 3 [Mus musculus]	0.04918	6.991365217
641,361	*Pinlyp*	ref|NP_001032220.1| phospholipase A2 inhibitor and Ly6/PLAUR domain-containing protein precursor [Mus musculus]	0.051329	7.831155673
68,311	*Lypd2*	ref|XP_006521375.1| PREDICTED: ly6/PLAUR domain-containing protein 2 isoform X1 [Mus musculus]	0.051329	7.835879412
22,160	*Twist1*	ref|NP_035788.1| twist-related protein 1 [Mus musculus]	0.063547	5.484675109
14,264	*Fmod*	ref|NP_067330.1| fibromodulin precursor [Mus musculus]	0.069982	6.543157283
11,752	*Anxa8*	ref|NP_038501.2| annexin A8 isoform 1 [Mus musculus]	0.076501	6.858814226
NA	LOC10816809*6*	ref|XP_017171171.1| PREDICTED: zinc finger protein 120-like [Mus musculus]	0.095397	6.307875483

**Table 3:** Top genes downregulated in the brain of male compared to female Amami spiny rats

Entrez ID	Gene Symbol	Gene Name	Adjusted P value	Fold Change
20,716	*Serpina3n*	ref|XP_011242348.1| PREDICTED: serine protease inhibitor A3N isoform X1 [Mus musculus]	0.004689083	−10.4865403440309
19,374	*Rag2*	ref|NP_033046.1| V(D)J recombination-activating protein 2 [Mus musculus]	0.01269514	−8.06797724828624
17,345	*Mki67*	ref|XP_006507476.1| PREDICTED: antigen KI-67 isoform X3 [Mus musculus]	0.019586606	−8.81256385444814
12,062	*Bdkrb2*	ref|XP_011242293.1| PREDICTED: B2 bradykinin receptor isoform X1 [Mus musculus]	0.031873832	−6.98198805080256

### Differential expression of transcripts in the brain of male and female Amami spiny rats

**Fig. 3:**
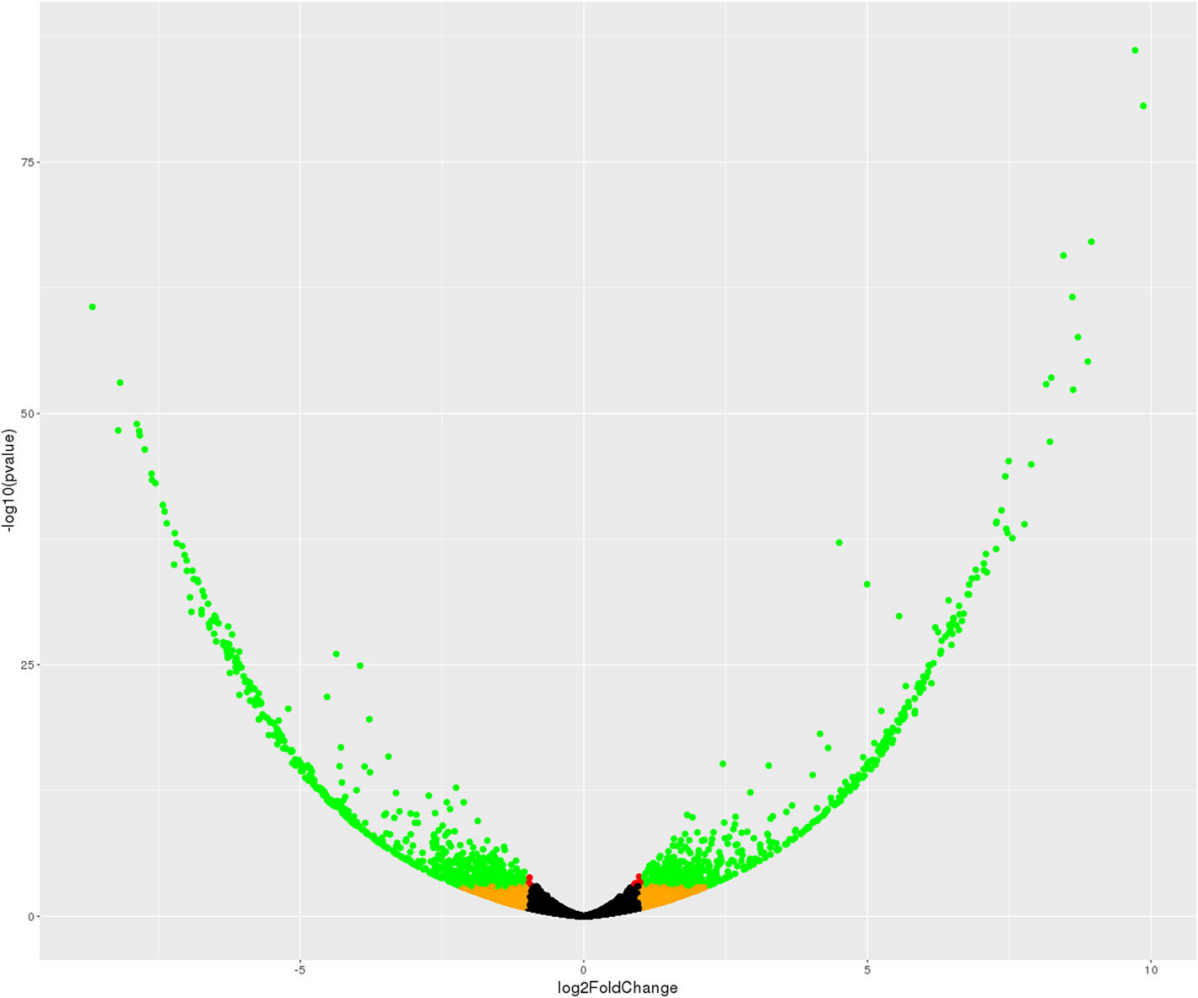
Volcano plot analyses of differentially expressed transcripts. The volcano plot represents the relationship of each transcript’s log2 fold change vs -log10 pvalue. Orange points represent expression ratios greater than 2X (either up or down), red points represent transcripts whose FDR-corrected p-values are < 0.05 and green points are transcript where both properties occur (fold-change > +/− 2X and FDR < 0.05)

Next, we considered brain transcripts that may show differential expression in the brain of male vs. female Amami spiny rats and vice versa. With this approach, 432 transcripts were upregulated in males and 508 transcripts are downregulated in males (volcano plot shown in Fig. 3; Additional file 2). A subset of transcripts upregulated in males is listed in Table 4. As shown, several transcripts from epigenetic-associated genes were upregulated in males, such as Sin3-Associated Protein P30-Like (*Sap30l*)*,* SET domain and mariner transposase fusion gene (*Setmar*)*,* methyl-CpG-binding domain protein 4 (*Mbd4*), and histone deacetylase 2 (*Hdac2*). Transcripts from genes encoding several zinc finger proteins were also increased in the brain of males compared to females. Some examples of these genes include zinc finger protein X-linked (*Zfx*), zinc finger and BTB domain-containing protein 44 (*Zbtb44*), zinc finger protein 708 (*Zfp708*), LOC102640673, zinc finger protein (*Zpr1*), and zinc finger protein 655 *(Zfp655*)*.* Transcripts from genes associated with male reproduction were also elevated in the brain of Amami spiny rat males, such as testis-expressed protein 9 (*Tex9)* and spermatogenesis Associated 7 (*Spata7*)*.*

**Table 4:** Select transcripts upregulated in the brain of male compared to female Amami spiny rats

Entrez ID	Gene Symbol	Gene Name	Adjusted P value	Fold Change
54,673	*Sh3glb1*	ref|NP_062337.1| endophilin-B1 isoform 2 [Mus musculus]	1.53E-83	864.5829107
78,521	*B230219D22Rik*	gb|AAH06718.1| B230219D22Rik protein [Mus musculus]	2.72E-79	983.6329675
NA	*NA*	gb|EDL18689.1| mCG11475, isoform CRA_b, partial [Mus musculus]	9.45E-57	569.9580734
224,143	*Poglut1*	ref|NP_001287756.1| protein O-glucosyltransferase 1 isoform 2 [Mus musculus]	1.27E-56	447.6240011
NA	*LOC110562326*	ref|XP_021514718.1| protein piccolo-like [*Meriones unguiculatus*]	9.48E-53	452.3360482
110,616	*Atxn3*	ref|NP_083981.2| ataxin-3 isoform 1 [Mus musculus]	4.64E-52	316.17416
20,687	*Sp3*	ref|NP_001018052.1| transcription factor Sp3 isoform 1 [Mus musculus]	2.06E-51	296.2983179
234,138	*Tti2*	ref|NP_659176.2| TELO2-interacting protein 2 isoform a [Mus musculus]	4.10E-44	254.9441833
67,763	*Prpsap1*	ref|XP_006534081.1| PREDICTED: phosphoribosyl pyrophosphate synthase-associated protein 1 isoform X3 [Mus musculus]	3.07E-39	173.6371454
21,778	*Tex9*	ref|XP_006511083.1| PREDICTED: testis-expressed sequence 9 protein isoform X2 [Mus musculus]	5.13E-38	162.8235175
12,237	*Bub3*	ref|NP_001304279.1| mitotic checkpoint protein BUB3 [Mus musculus]	5.13E-38	218.263186
64,297	*Gprc5b*	ref|NP_071865.1| G-protein coupled receptor family C group 5 member B isoform 2 precursor [Mus musculus]	5.61E-38	190.0165701
109,229	*Fam118b*	ref|XP_006510001.1| PREDICTED: protein FAM118B isoform X2 [Mus musculus]	6.15E-36	167.69919
NA	*WDR48*	CAB82402.1 WD repeat domain 48 [Homo sapiens]	4.53E-35	144.6287527
NA	*NA*	gb|EDL35184.1| mCG124360, partial [Mus musculus]	1.18E-34	22.66124115
19,280	*Ptprs*	ref|XP_006523939.1| PREDICTED: receptor-type tyrosine-protein phosphatase S isoform X2 [Mus musculus]	2.83E-34	142.1259243
70,408	*Polr3f*	ref|NP_084039.2| DNA-directed RNA polymerase III subunit RPC6 [Mus musculus]	9.10E-34	143.627746
26,440	*Psma1*	ref|NP_036095.1| proteasome subunit alpha type-1 [Mus musculus]	9.10E-34	151.24105
235,132	*Zbtb44*	ref|NP_001108602.1| zinc finger and BTB domain-containing protein 44 isoform a [Mus musculus]	2.01E-33	127.0100843
104,871	*Spata7*	ref|NP_849245.2| spermatogenesis-associated protein 7 homolog isoform 1 [Mus musculus]	0.003792691	42.00659874
50,724	*Sap30l*	ref|NP_001074637.1| histone deacetylase complex subunit SAP30L [Mus musculus]	2.12E-05	11.07274329
432,769	*Zfp708*	ref|NP_001278995.1| zinc finger protein 708 isoform d [Mus musculus]	0.000446448	3.198427211
102,640,673	LOC102640673	ref|XP_011245044.1| PREDICTED: zinc finger protein 431-like [Mus musculus]	0.00055526	2.333373335
22,687	*Zpr1*	ref|NP_035882.1| zinc finger protein ZPR1 [Mus musculus]	0.000712498	3.572041181
74,729	*Setmar*	ref|NP_848478.2| histone-lysine N-methyltransferase SETMAR isoform 1 [Mus musculus]	0.007934623	2.434879906
72,611	*Zfp655*	ref|XP_017176604.1| PREDICTED: zinc finger protein 655 isoform X2 [Mus musculus]	0.009577135	2.092891524
17,193	*Mbd4*	ref|NP_034904.2| methyl-CpG-binding domain protein 4 [Mus musculus]	0.01479798	2.242327622
22,764	*Zfx*	ref|XP_006528028.1| PREDICTED: zinc finger X-chromosomal protein isoform X1 [Mus musculus]	0.036600879	2.600771985

Examples of transcripts that were downregulated in males (or upregulated in females) are listed in Table 5. Unlike those that were upregulated in males, these transcripts are derived from genes representing diverse functions, although select examples of epigenetic-associated genes are included on this list, e.g. lysine acetyltransferase 6A-like (*Kat6al*), and zinc finger proteins (e.g., zinc finger protein 410 [*Zfp410*]*).* The list of the top 35 transcripts upregulated in females includes the associated genes for, mitofusin 1 (*Mfn1*) and mitofusin 2 (*Mfn2*), which encode for GTPases in the outer membrane of the mitochondria. The list also includes transcripts from the genes cullin 1 (*Cul1*) and cullin-3 (*Cul3*), which encode hydrophobic proteins that provide a scaffold for post-translational modification of cellular proteins by ubiquitin ligases (E3). Transcripts derived from several ubiquitin ligase genes were upregulated in the brain of female Amami spiny rats (ring finger protein 220 [*Rnf220*], SNF2 histone linker PHD RING helicase [*Shprh*], Praja ring finger ubiquitin ligase 2 [*Pja2*], Siah E3 Ubiquitin Protein Ligase 2 [*Siah2*], Mahogunin ring finger 1 [*Mgrn1*], and ring finger protein 25 [*Rnf25*]).

**Table 5:** Select transcripts down-regulated in the brain of male compared to female Amami spiny rats

Entrez ID	Gene Symbol	Gene Name	Adjusted P value	Fold Change
213,081	*Wdr19*	ref|XP_011239015.1| PREDICTED: WD repeat-containing protein 19 isoform X1 [Mus musculus]	7.18E-59	−205.685732956079
100,862,175	*Gm10698*	ref|XP_003945495.1| PREDICTED: transmembrane emp24 domain-containing protein 2 [Mus musculus]	1.20E-47	−196.721787522148
110,616	*Atxn3*	ref|NP_083981.2| ataxin-3 isoform 1 [Mus musculus]	2.28E-47	−176.574809890753
67,398	*Srpr*	ref|NP_080406.1| signal recognition particle receptor subunit alpha [Mus musculus]	1.01E-46	−173.160534061175
52,708	*Zfp410*	ref|XP_006516149.1| PREDICTED: zinc finger protein 410 isoform X1 [Mus musculus]	2.48E-46	− 156.169760821545
215,015	*Fam20b*	ref|XP_006496800.1| PREDICTED: glycosaminoglycan xylosylkinase isoform X1 [Mus musculus]	5.95E-45	− 153.092554360035
26,554	*Cul3*	ref|NP_057925.1| cullin-3 isoform 1 [Mus musculus]	1.10E-42	−142.068207466613
217,463	*Snx13*	ref|NP_001014973.2| sorting nexin-13 [Mus musculus]	4.19E-42	−138.676695678961
73,834	*Atp6v1d*	ref|NP_076210.1| V-type proton ATPase subunit D [Mus musculus]	8.90E-42	−135.2167097375
23,996	*Psmc4*	ref|NP_036004.2| 26S protease regulatory subunit 6B [Mus musculus]	4.30E-39	−125.773520519436
66,049	*Rogdi*	ref|NP_573448.2| protein rogdi homolog [Mus musculus]	4.88E-38	−124.62265035744
93,687	*Csnk1a1*	ref|XP_006526451.1| PREDICTED: casein kinase I isoform X7 [Mus musculus]	5.23E-37	−118.167538690104
170,731	*Mfn2*	ref|XP_017175503.1| PREDICTED: mitofusin-2 isoform X2 [Mus musculus]	4.59E-36	−117.421102051596
20,515	*Slc20a1*	ref|XP_011237701.1| PREDICTED: sodium-dependent phosphate transporter 1 isoform X1 [Mus musculus]	1.14E-35	−136.72988988069
75,221	*Dpp3*	ref|XP_006531917.1| PREDICTED: dipeptidyl peptidase 3 isoform X1 [Mus musculus]	7.59E-35	−111.072178294408
106,763	*Ttbk1*	ref|XP_006523508.1| PREDICTED: tau-tubulin kinase 1 isoform X1 [Mus musculus]	2.45E-34	−109.071601645524
13,030	*Ctsb*	ref|NP_031824.1| cathepsin B preproprotein [Mus musculus]	2.67E-33	−116.144236667802
217,463	*Snx13*	ref|XP_006515125.2| PREDICTED: sorting nexin-13 isoform X1 [Mus musculus]	1.45E-32	−100.803592377535
67,095	*Trak1*	ref|NP_780323.2| trafficking kinesin-binding protein 1 [Mus musculus]	2.36E-32	−106.108251172079
26,965	*Cul1*	ref|NP_036172.1| cullin-1 [Mus musculus]	3.68E-32	−98.8568050703889
54,380	*Smarcal1*	ref|XP_006496208.1| PREDICTED: SWI/SNF-related matrix-associated actin-dependent regulator of chromatin subfamily A-like protein 1 isoform X3 [Mus musculus]	1.95E-31	−205.685732956079
103,466	*Nt5dc3*	ref|NP_780540.2| 5′-nucleotidase domain-containing protein 3 [Mus musculus]	2.84E-31	−196.721787522148
382,030	*Cnep1r1*	ref|NP_083350.2| nuclear envelope phosphatase-regulatory subunit 1 [Mus musculus]	8.86E-31	−176.574809890753
235,493	LOC108642943	ref|XP_006746095.1| PREDICTED: histone acetyltransferase KAT6A-like [*Leptonychotes weddellii*]	1.26E-28	−173.160534061175
NA	*Rab1a*	ref|NP_033022.1| ras-related protein Rab-1A [Mus musculus]	1.48E-28	−156.169760821545
19,324	*Neo1*	ref|NP_032710.2| neogenin isoform 1 precursor [Mus musculus]	5.29E-27	−153.092554360035
77,097	*Tanc2*	ref|XP_006534538.1| PREDICTED: protein TANC2 isoform X4 [Mus musculus]	2.58E-26	−88.0388621340817
74,011	*Slc25a27*	ref|XP_017173171.1| PREDICTED: mitochondrial uncoupling protein 4 isoform X7 [Mus musculus]	3.00E-26	−74.4613531649071
67,414	*Mfn1*	ref|NP_077162.2| mitofusin-1 [Mus musculus]	4.32E-26	−80.4285914968941
66,743	*Rnf220*	ref|NP_080015.3| E3 ubiquitin-protein ligase Rnf220 isoform 1 [Mus musculus]	2.38E-13	−31.0675077000804
268,281	*Shprh*	ref|XP_006512803.1| PREDICTED: E3 ubiquitin-protein ligase SHPRH isoform X1 [Mus musculus]	5.88E-10	−22.3942689224801
224,938	*Pja2*	ref|NP_659108.1| E3 ubiquitin-protein ligase Praja-2 isoform b [Mus musculus]	1.81E-09	−19.4062860871404
20,439	*Siah2*	ref|NP_033200.2| E3 ubiquitin-protein ligase SIAH2 [Mus musculus]	1.02E-06	−8.29311442392182
17,237	*Mgrn1*	ref|XP_006521895.1| PREDICTED: E3 ubiquitin-protein ligase MGRN1 isoform X1 [Mus musculus]	1.38E-06	−6.25228780529387
57,751	*Rnf25*	ref|NP_067288.2| E3 ubiquitin-protein ligase RNF25 isoform 1 [Mus musculus]	0.026566445	−2.67258820899355

### Gene interaction metanalyses and pathway analyses

**Fig. 4:**
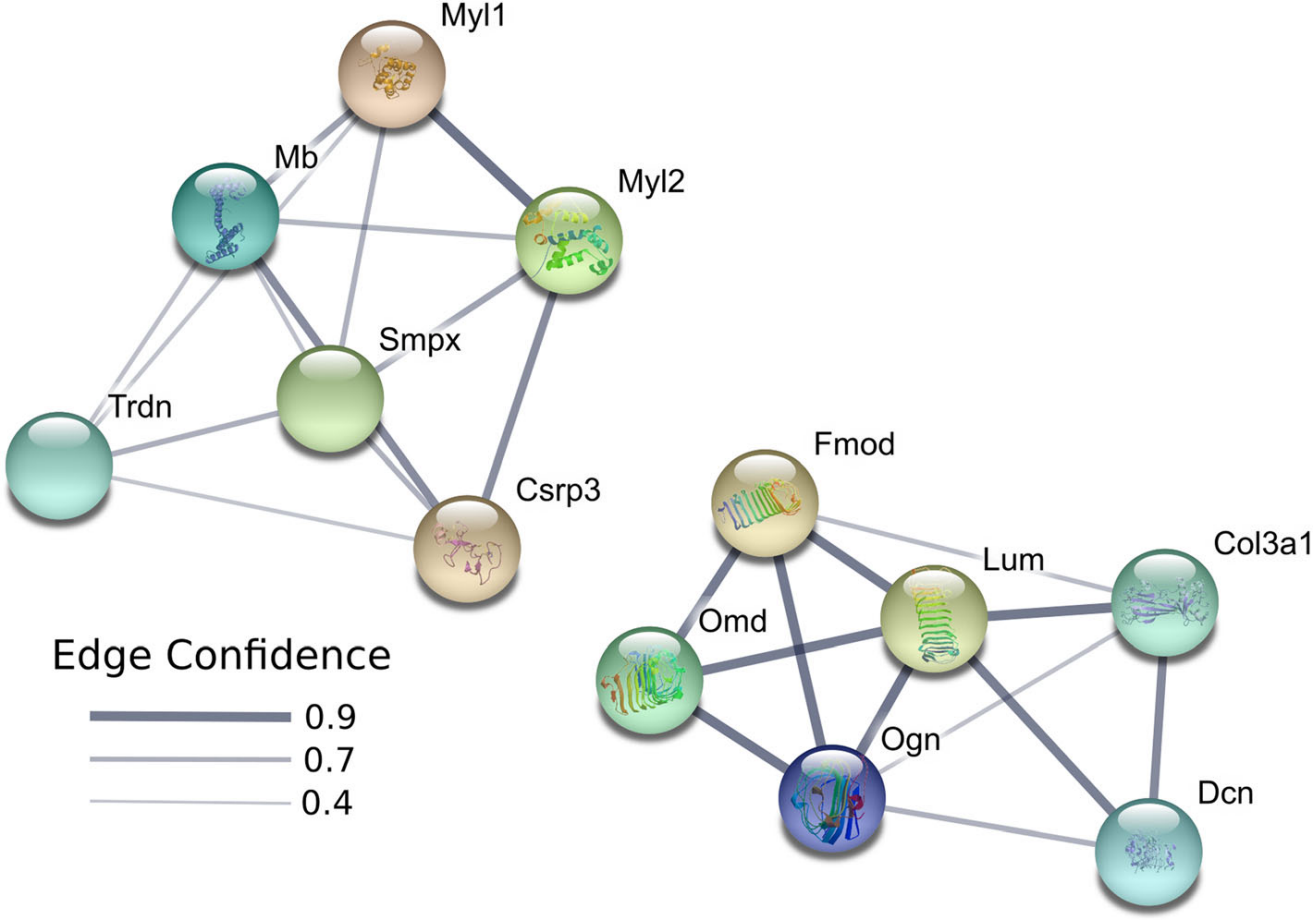
STRING pathway analyses diagram for genes upregulated in males. Based on genes identified to be upregulated in males, two main clusters are identified with one cluster comprised of genes such as *Myl1*, *Mb*, *Trdn*, *Myl2*, *Csrp3*, and *Smpx* in the center. The second cluster includes *Fmod*, *Omd*, *Ogn*, *Dcn*, *Col3a1*, and *Lum*. Network nodes represent proteins. Splice isoforms or post-translational modifications are collapsed, i.e. each node represents all the proteins produced by a single, protein-coding gene locus. Node colors are arbitrary. Edges represent protein-protein associations. Associations are meant to be specific and meaningful, i.e. proteins jointly contribute to a shared function; this does not necessarily mean they are physically binding each other. Edge confidence is the relative amount of supporting evidence for the connection between two proteins in the diagram. Thicker lines have more support; thinner lines have less support

We used STRING [56] to identify potential interactions between differentially expressed genes. Based on the genes identified to be upregulated in males, two main STRING clusters were identified with one cluster comprised of genes such as myosin light chain 1 (*Myl1*), myoglobin (*Mb*), triadin (*Trdn*), myosin regulatory light chain 2 (*Myl2*), cysteine and glycine rich protein 3 (*Csrp3*), and small muscle protein X-linked (*Smpx*) in the center (Fig. 4). The second cluster includes fibromodulin (*Fmod*), osteomodulin (*Omd*), osteoglycin (*Ogn*), decorin (*Dcn*), collagen type III alpha 1 chain (*Col3a1*), and *Lum*. Pathways that were enriched for genes upregulated in males include collagenesis, myofibril formation, and proteinaceous extracellular matrix (Additional file 3). The four genes (*Mki67*, *Bdkrb2*, *Rag2*, and *Serpina3n*) upregulated in females do not share any similar pathways, and thus, do not form a distinct cluster.

**Fig. 5:**
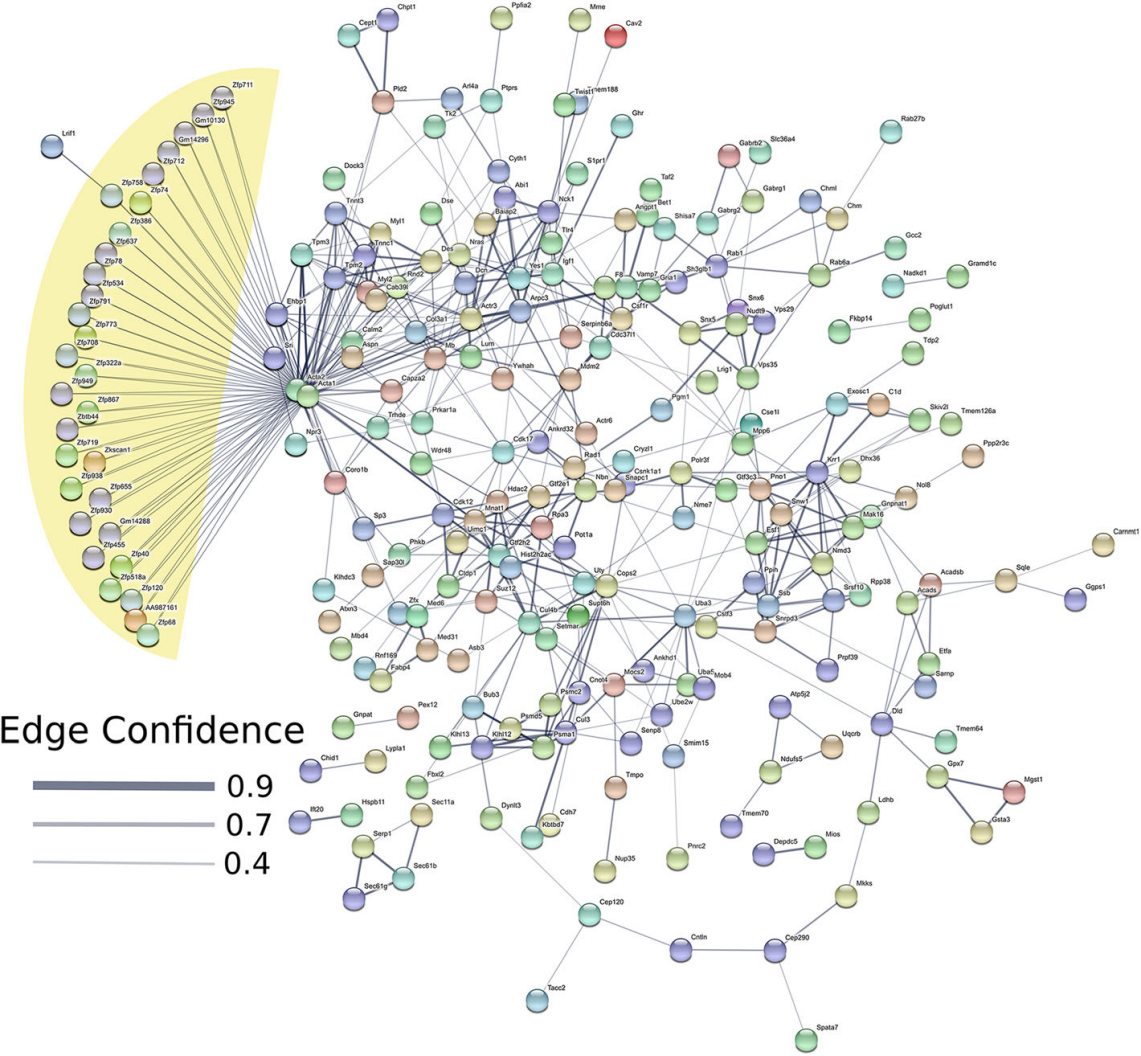
STRING pathway analyses diagram for transcripts upregulated in males. The primary group of transcripts upregulated in the brain of male Amami spiny rat are transcripts encoding zinc finger proteins (yellow highlighted area). Several other smaller clusters are present. Network nodes represent proteins. Splice isoforms or post-translational modifications are collapsed, i.e. each node represents all the proteins produced by a single, protein-coding gene locus. Node colors are arbitrary. Edges represent protein-protein associations. Associations are meant to be specific and meaningful, i.e. proteins jointly contribute to a shared function; this does not necessarily mean they are physically binding each other. Edge confidence is the relative amount of supporting evidence for the connection between two proteins in the diagram. Thicker lines have more support; thinner lines have less support

When individual transcripts are considered, the primary group that is upregulated in the brain of male Amami spiny rat are zinc finger proteins (Fig.5, yellow highlighted area). Several other smaller clusters are present. Pathways that are enriched in transcripts upregulated in males include Krueppel-associated box and zinc finger pathways (zinc finger zinc finger, C2H2-type/integrase DNA, zinc finger, C2H2, and zinc finger C2H2-like (Additional file 4). Processes associated with upregulated male transcripts include, metabolism, tissue development, regulation of ATPase activity, regulation of several aspects of organelle function, nucleoplasm, microtubule cytoskeleton, and GABA-A receptor complex (Additional file 4).

**Fig. 6:**
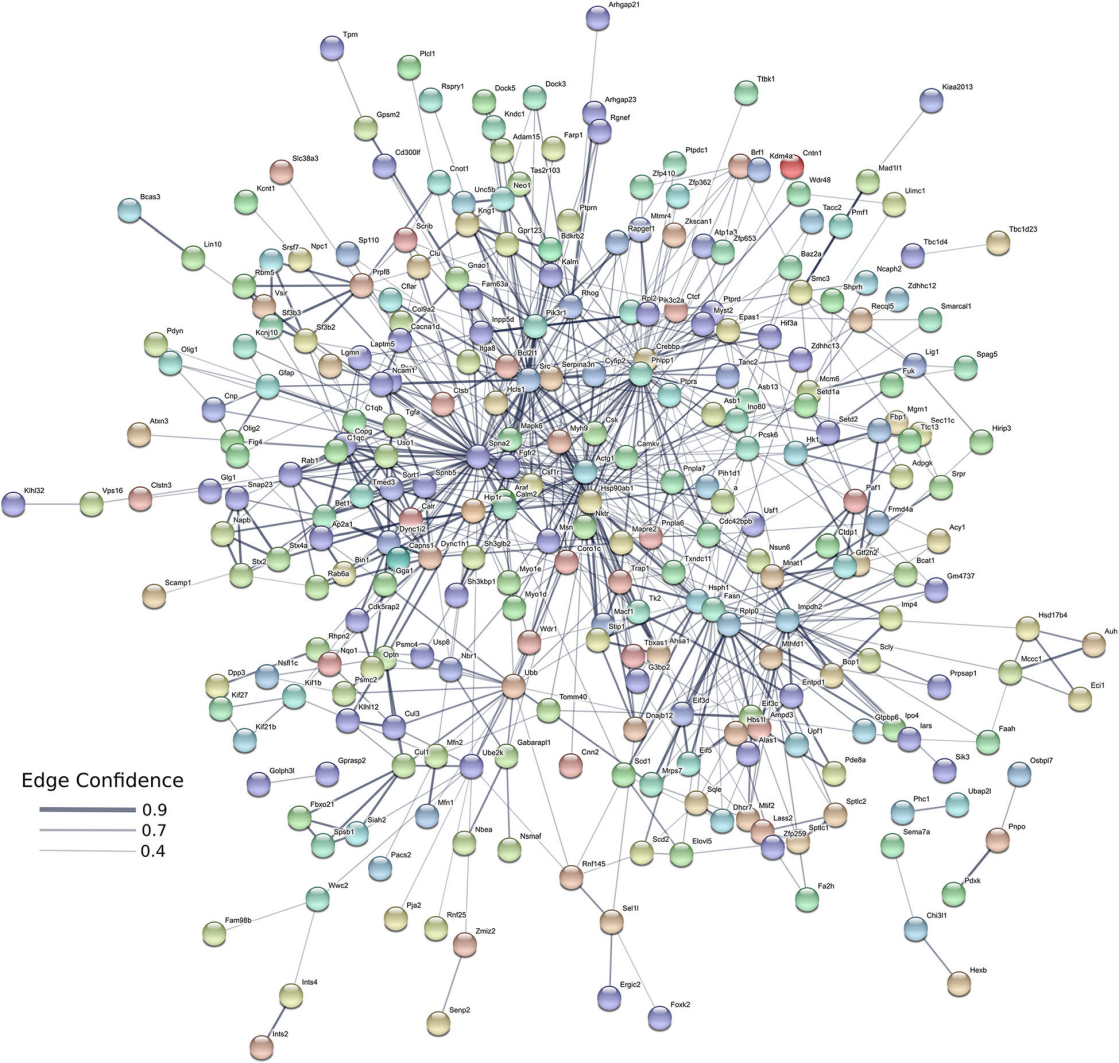
STRING pathway analyses diagram for transcripts upregulated in females. Based on the elevated transcripts, several pathways are affected in females. Examples include nucleotide, purine ribonucleoside, ribonucleotide, carbohydrate derivative, heterocyclic, zinc ion, ATP, transitional metal ion, cytoskeletal protein, GABA receptor binding pathways, hydrolase, nucleoside triphosphate, ATPase, L-amino acid transmembrane transporter, and palmitoyl-CoA 9-desaturase activity. Network nodes represent proteins. Splice isoforms or post-translational modifications are collapsed, i.e. each node represents all the proteins produced by a single, protein-coding gene locus. Node colors are arbitrary. Edges represent protein-protein associations. Associations are meant to be specific and meaningful, i.e. proteins jointly contribute to a shared function; this does not necessarily mean they are physically binding each other. Edge confidence is the relative amount of supporting evidence for the connection between two proteins in the diagram. Thicker lines have more support; thinner lines have less support

The transcripts upregulated in females represent diverse pathways (Fig. 6, Additional file 5), including nucleotide, purine ribonucleoside, ribonucleotide, carbohydrate derivative, heterocyclic, zinc ion, ATP, transitional metal ion, cytoskeletal protein, and GABA receptor binding. Additional pathways include hydrolase, nucleoside triphosphate, ATPase, L-amino acid transmembrane transporter, and palmitoyl-CoA 9-desaturase activity.

### qPCR analyses

**Fig. 7:**
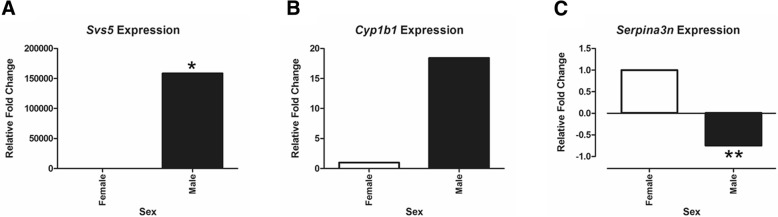
qPCR analyses for select genes in the brain of female and male Amami spiny rats. A) *Svs* expression. B) *Cyp1b1*. C) *Serpina3n*. *p = 0.0005. **p = 0.05

Due to limited remaining tissue, only three candidate genes shown to be altered in the RNAseq analyses were validated with qPCR. These include two that were upregulated in males: *Svs5* and *Cyp1b1* and one that was upregulated in females: *Serpina3n*. The three genes chosen all demonstrated significant adjusted *p* values and fold change and have previously ascribed roles in guiding sexual differentiation and/or neural function. As shown in Fig. 7, *Svs5* expression was considerably upregulated in the brain of males compared to females (*p* = 0.0005); whereas *Serpina3n* was significantly down-regulated in males vs. females (*p* = 0.05), which is identical to the profile detected with RNAseq. While *Cyp1b1* showed a tendency to be increased in males, as seen with RNAseq, the qPCR results were not significant.

## Discussion

When SRY was identified, it was presumed that it regulated sexual differentiation in all eutherian mammals [14–18]. However, those species that defy our expectations reveal the complexity and elegance of sexual differentiation that like other biological processes has been subject to evolutionary forces million years in the making. Such is the case for select species within *Tokudaia* and *Ellobius*. Intriguingly, 46, XX human male brothers lacking SRY possess testes but are azoospermic [57]. Overexpression of SOX9 in these SRY-negative men likely stimulates male sexual differentiation [58, 59]. Thirty-eight intersex, XX SRY-negative cases have also been reported in pigs [60].

How sexual differentiation occurs in rodent species where males lack a Y chromosome and SRY remains enigmatic. In Amami spiny rats, two possible candidates have been postulated to regulate testes formation: *Cbx2*, which acts upstream of *Sry*, and *Er71* [42, 43]. The wave of sexual differentiation affects both the gonad and brain [1–3]. While in the latter steroid hormones, testosterone and estrogen, are the guiding factors, SRY might also be involved [61]. In those species that possess SRY, it continues to be expressed in the adult male brain [62, 63]. At the current time, we cannot obtain embryonic or neonatal brain samples from Amami spiny rat to identify putative sex determining gene(s)/transcript(s) in this species. Our prediction at the outset was that similar to mice, birds, and humans [44–48], sex differences would be identified in gene/transcript expression in adult Amami spiny rat, albeit many of these many not necessarily be sex-determining genes. The additional objective of the current work was to establish the first reference transcriptomic library for Amami spiny rat that would likely facilitate future work seeking to identify those gene(s)/transcript(s) compensating for the absence of the Y chromosome and SRY in this species. With these limitations and potential strengths in mind, we analyzed the transcriptomes of the adult male and female Amami spiny rat brains to determine which genes and transcripts show sexually dimorphic patterns of expression.

While certain genes differed in expression between male and female Amami spiny rat, as detailed further below, more prominent changes were discovered when comparing sex differences of individual transcripts. The aggregate characteristics of the differentially expressed transcripts are compelling. While the proportional contribution of each splice variant to the total mRNA for a given gene might be low, such variants might demonstrate different potencies in binding and activating various cellular pathways. Studies with mice and other taxa demonstrate that expression of such transcript variants can result in dramatic sex differences and potentially underpin sexual differentiation [64–67]. In Amami spiny rat, additional phenotypic studies are needed to validate the significance of these findings and whether they broadly differ across various sexually dimorphic organs, including the gonad, and discrete brain regions.

Several transcripts upregulated in the whole brain of males compared to females encode zinc finger proteins. Additionally, gene set enrichment analyses of the genes from which the upregulated transcripts derive identified several zinc-finger related pathways, including Krueppel-associated box (KRAB). KRAB only (KRAB-O) serves as an SRY-interacting protein [68, 69]. Zinc finger proteins are polypeptides exhibiting sequence-specific, nucleic acid-binding role, and serve as trans-acting molecules they govern cellular growth and differentiation. Zinc finger protein Y-linked (*Zfy*) is another gene located on the Y chromosome, and while not the TDF, it is essential for normal spermatogenesis [70], although an isolated human case has been reported of normal sexual differentiation in the absence of ZFY [71]. Similar to SRY, this gene continues to be expressed in the brain of adult men [62]. In the Amami spiny rat, *Zfy*, along with eukaryotic translation initiation factor 2 subunit 3, Y-linked (*Eif2s3y*) and lysine Demethylase 5D (*Kdm5d*), have been translocated to the X chromosome [72]. The current brain transcriptome studies detected *Zfy*, but the gene and individual transcripts showed similar expression pattern in males and females.

A corollary gene is present on the X chromosome, *Zfx* with male mice harboring a mutation of this gene undergoing normal gonadal and male genitalia sexual differentiation but showing markedly reduced sperm counts [73]. Mutant *Zfx* female mice possess normal ovaries and external genitalia but show diminished oocyte populations and reduced fecundity culminating in abbreviated reproductive potential. Primordial germ cell populations prior to genital ridge migration are reduced in both XX and XY mice offspring and growth impairments are evident in both sexes. The collective data suggest that *Zfx* might thus be important in both male and female sexual differentiation in eutherian mammals. In *Drosophila*, the hermaphrodite (her) gene encodes for a C2H2-type zinc finger protein that demonstrates similar properties as *Zfy* and *Zfx* and presumably regulates terminal function in sexual differentiation [74]. In the current studies, one form *Zfx* was shown to be upregulated in the brain of males. However, several other genes encoding zinc finger proteins were also upregulated in males. Thus, we postulate that upregulation of *Zfx* acting possibly in concert with other C2H2-type zinc finger transcript forms in male Amami spiny rat might compensate for the absence of SRY and serve as the collective factors stimulating male sexual differentiation.

In support of this hypothesis, other zinc finger proteins exert essential sexual differentiation roles in lower animal species and mice after initial sexual differentiation. For instance, the flexuosa (fle1), encoding aC2H2 zinc finger protein, is an important repressor of female sexual differentiation in *Podospora anserina* [75]. The byr3 zinc finger protein (byr3) gene encoding a protein with seven finger domains affects sexual differentiation pathways in *Schizosaccharomyces pombe* [76]. In the Japanese wrinkled frog (*Rana rugosa*), an increase in testosterone causes sex reversal from female to male and accompanying decreases in zinc finger proteins, zinc finger protein 64 (*Zfp64*) and zinc finger protein 112 (*Zfp112*) [77]. In sex-reversed and normal mice, zinc finger protein 35 (*Zfp35*) is essential for normal spermatogenesis [78, 79]. Zinc finger proteins are elevated in human testicular cell lines overexpressing SRY, further highlighting the potential role of such proteins in maintaining sexual differentiation [80].

Sex differences in transcript isoforms encoding zinc finger proteins and other transcripts might originate due to epigenetic variation. In this aspect, intragenic DNA methylation and histone protein modifications can affect RNA polymerase II activity and thereby stimulate creation of alternative splice forms [81–85]. Heterochromatin protein 1 (HP1) might also play a role in DNA methylation effects on mRNA splicing [86]. DNA methylation and histone protein changes are evident in turtle species demonstrating temperature sex determination [87–91]. In the case of doublesex and mab-3 related transcription factor 1 (*Dmrt1*), DNA methylation status tightly associates with temperature and may thus be the master male sex determining gene in red-eared slider turtles (*Trachemys scripta*) [87].

RNAseq analyses revealed other genes and transcripts that were differentially expressed in the brain of males and females. It is not clear if such genes contribute to the initial brain sexual differentiation process or maintaining sex differences. Due to limited tissue, we could only validate three differentially expressed genes that were chosen based on there highly significant adjusted *p* value and overall fold change: *Svs5, Serpina3n*, and *Cyp1b1*. Of these, *Svs5* and *Serpina3n* expression patterns were identical to that observed with RNAseq with an increase in males and females, respectively. Further work is needed to determine the significance of these and other gene expression changes.

The current studies with adult Amami spiny rat yielded less genes that show sex-dependent brain expression than past studies with mice, birds, and humans [44–48]. This difference could be attributed to the fact that from an algorithmic standpoint, potentially high level of intra-sample variability would decrease the number of inter-sample genes whose differential expression fell below a FDR of 0.05. The current work presumably identified those genes that show the greatest differential expression between sample types and are potentially the most important.

One of the primary limitations of this study is that in this endangered species, we could only screen random brain tissues in limited number of adult male and female Amami spiny rats. It is not clear if genes and transcripts guiding initial brain sexual differentiation continue to be expressed. The fact that ZFY and SRY are expressed in the brain of adult men shores up this possibility [62, 63]. Usage of induced pluripotent stem cells (iPSC) generated from this species might be useful in validating the current findings [92, 93]. Future work should compare current RNAseq results in the brain of Amami spiny rat to those obtained in *Tokudaia* species that possess sex chromosomes, e.g., Okinawa spiny rat (*Tokudaia muenninki*) [94–96], as this approach may reveal those genes/transcripts essential in sex determination in Amami spiny rat. Should the population of these *Tokudaia* species recover and/or permission be granted in the future to attempt to breed them in captivity, follow-up transcriptome studies should be conducted with discrete brain regions identified to show sex differences in other species, such as the hypothalamus, and with individuals spanning from the embryonic to adult period. As detailed in the methods, one of the other limitations of this study is that it depends upon whole brain samples from animals who died of accidental causes during a mongoose eradication project. In these cases, brain and other tissues were collected several hours to half a day post-mortem. Thus, these studies should be considered a first pass attempt to obtain a reference transcriptome for this species and examine for sex-dependent differences in gene and transcript expression in the brain of adult individuals. A better understanding of underpinning biological mechanisms in this species may aide in recovery efforts to the point where sufficient animals can be bred in captivity and more precise studies in the brain, gonad, and other sex-dependent organs performed.

## Conclusions

In conclusion, transcriptomic analyses in the brain of adult male and female Amami spiny rats shows that sex differences are observed in select genes with more being upregulated in males than females. Comparison of individual transcript forms between males and females yielded more differences and potentially might provide mechanistic insight into how male sexual differentiation occurs in this Y chromosome deficient and SRY-negative rodent species. The most attractive candidates are upregulation of a variety of transcript forms encoding zinc finger proteins, including *Zfx.* Such sex differences in these and other alternative splice forms might originate due to epigenetic modifications. It remains to be determined if any of these identified gene(s)/transcript(s) modulate initial sexual differentiation in this species.

## Methods

### Animal tissue collection and RNA isolation

*T. osimensis* is endangered (The IUCN Red List of Threatened Species; https://www.iucnredlist.org/). This species has been protected by the Japanese government as a natural monument since 1972 and Nationally Endangered Species of Wild Fauna and Flora since 2016. With permission from the Agency for Cultural Affairs and the Ministry of the Environment in Japan, tissues were harvested from animals who died accidentally as part of a Japanese government approved mongoose eradication project on the island this species inhabits. A copy of the government approval, which has also been translated into English, to harvest brain and other tissues from animals that die accidentally has been provided to the journal.

The brain and other tissues were harvested and frozen several hours to half a day post-mortem. Brain tissues were scraped by a needle or tweezer through the posterior cranial fossa in order to preserve the skull, which is also rare and needed for taxonomic studies. Several portions of brain tissue were then mixed and frozen together. Frozen male (*n* = 3) and female (*n* = 4) brain samples stored at − 80 °C were placed in RNA*later*™-ICE Frozen Tissue Transition Solution (ThermoFisher Scientific, Waltham, MA) and stored overnight at − 20 °C per the manufacture’s protocol. The samples were then shipped to Dr. Rosenfeld’s laboratory at the University of Missouri. RNA was isolated from each brain sample with TRIzol Plus RNA Purification Kit (ThermoFisher Scientific). The quantity and quality of the RNA was determined with a Nanodrop ND1000 spectrophotometer (Nanodrop Products, Wilmington, DE). The results were further confirmed by analyzing the RNA on the Fragment Analyzer (Advanced Analytical Technologies, Ankeny, IA) where the samples showed RIN values ranging from 5 to 7 with only slight RNA degradation. Unfortunately, as this species is endangered and the government will only permit tissues to be harvested from animals that died accidentally, this is currently the best material that can be obtained.

### Illumina TruSeq RNA library preparation and sequencing

Libraries were constructed following the manufacturer’s protocol with reagents supplied in Illumina’s TruSeq mRNA Stranded Library Preparation Kit. Briefly, the poly-A containing mRNA was purified from total RNA, mRNA was fragmented, double-stranded cDNA generated from fragmented RNA, and the index containing adapters were ligated to the ends.

The final construct of each purified library was evaluated using the Fragment Analyzer (Advanced Analytical Technologies, Ankeny, IA) automated electrophoresis system, quantified with the Qubit flourometer using the quant-iT HS dsDNA reagent kit (Invitrogen), and diluted according to Illumina’s standard sequencing protocol for sequencing on the NextSeq 500. Libraries were sequenced at the University of Missouri DNA Core Facility to obtain 75 base pair, single end reads.

### Bioinformatics analyses

Since the Amami spiny rat genome has not been sequenced or annotated, we had to develop our own reference transcriptomic library. Previously described methods [97] were used to assemble a de novo reference transcriptome and analyze differential expression. Raw RNA-Seq reads were subjected to a multi-phase quality control regimen as previously described [98]. Specifically, 50-mer RNA-Seq reads from the Illumina HiSeq were first cleaned using the Fastx Toolkit (https://github.com/agordon/fastx_toolkit) to trim the 3′ ends of low quality (phred score < 20) bases and dropping reads when less than 32 bases remaining. Reads were then filtered to exclude those that did not have a minimum of 95% of their bases with a quality score of 20 or more. Adapter sequence was removed with CutAdapt, version 1.8.3 [99]. To create a final set of quality-controlled RNA-Seq reads, hereafter referred to as QC reads, foreign or undesirable reads were removed by sequence matching to the Phi-X genome (NC_001422.1) [100], the relevant ribosomal RNA genes as downloaded from the National Center for Biotechnology Information [101] or rodent repeat elements in RepBase, version 20.02 [102] using Bowtie, version 1.1.1 [103]. QC reads from all samples were used to generate a reference transcriptome assembly (RTA) by using Trinity, version 2.4.0, and default settings, except for random-access memory (RAM) and central processing unit (CPU) parameters [104]. Subsequently, QC reads from each sample were aligned to the RTA and expression estimates for each transcript > 200 nt in length were determined using RSEM via the align_and_estimate_abundance.pl script within the Trinity suite and these options: --prep_reference --est_method RSEM --aln_method bowtie2 --fragment_length 375 --fragment_std 40 --trinity_mode. The transcript expression estimates were normalized and prepared for downstream analysis using the abundance_estimates_to_matrix.pl script within the Trinity suite and these options: --est_method RSEM --cross_sample_norm TMM. Samples were subjected to a differential expression (DE) analysis using tximport [54] and DESeq2 [55]. Only transcripts having a sum of counts from all samples greater than 25 were analyzed. A transcript or gene was considered DE if the false discovery rate (FDR) associated with its expression ratio between conditions was less than 0.05. For enrichment, pathway and network analyses, we used various R packages and web sites, including EGSEA [105], DAVID [106, 107], TargetMine [108] and STRING [56]. Prior to using these programs, gene names were assigned to each DE transcript based on a BLASTX best hit strategy. Briefly, a gene name was assigned to a transcript if its best BLASTX alignment to either a human, rat or mouse protein carried an E-value of less than 1e^− 06^ and the aligned regions covered at least 40% of the reference protein. This protocol allowed us to assign gene names to approximately 65% of the differentially expressed transcripts. Assembly metrics were generated using TransRate [109] using the --assembly option and BUSCO, v3, using the --mode transcriptome option and the vertebrate database [49]. To analyze for transcript differences, we used the abundance_estimates_to_matrix.pl script included with the Trinity software to generate a matrix of counts for each transcript using the RSEM method. Subsequently, we used the run_DE_analysis.pl script included with the Trinity software to determine differentially expressed transcripts using the DESeq2 method. For each analysis, we used the list of differentially expressed (q < 0.05) gene symbols as input and with default options for the particular piece of software.

PCA plots were generated for genes and transcripts that showed sex dependent differences. The first and second principal components (which contain the two highest proportions of variation) were plotted on the X and Y axes, respectively. We used normalized gene expression estimates generated by the Trinity script abundance_estimates_to_matrix.pl using the TMM (Trimmed Mean of M values, [110]) normalization method.

### qPCR analyses

Total RNA was reverse transcribed into cDNA using the QuantiTect Reverse Transcription Kit (Catalogue #205310, Qiagen). The quantitative polymerase chain reaction (qPCR) procedure was performed on the Applied Biosystems 7500 Real-Time PCR System (Carlsbad, CA) using the QuantiTect SYBR Green PCR Kit (Catalogue #204143; Qiagen). Primer sequences for the genes examined are listed in Additional file 6: Table S1, and primers were purchased from IDT (Coralville, IA). The qPCR conditions employed were 1) 15 min at 95 °C for polymerase activation 2) 40 cycles of: denaturation 40 s at 94 °C, annealing 40 s at 55 °C, and extension 72 °C for 1.50 min 3) Dissociation melt curve analysis from 60 °C to 90 °C. The internal control primer was glyceraldehyde-3-phosphate dehydrogenase (*Gapdh)* and test genes included: *Svs5, Cyp1b1, and Serpina3n*.

### Statistics

The qPCR Data were analyzed by using GraphPad Prism 5 (GraphPad Software, La Jolla, CA). ANOVA was done to compare delta cycle threshold (dCt) values obtained in the brain samples of males to those obtained from female brain samples. However, in Fig. 6, data are expressed as relative fold change based on the 2^-ΔΔCt^ method.

## Additional files

Additional file 1: Gene expression difference in the brain of male vs. female Amami spiny rats. (XLSX 29 kb)

Additional file 2: Transcript isoforms differentially regulated in the brain of male vs. female Amami spiny rats. (XLSX 529 kb)

Additional file 3: Pathways upregulated in male Amami spiny rats based on differential gene expression. (XLSX 10 kb)

Additional file 4: Pathways upregulated in male Amami spiny rats based on differential transcript isoform expression. (XLSX 30 kb)

Additional file 5: Pathways upregulated in female Amami spiny rats based on differential transcript isoform expression. (XLSX 62 kb)

Additional file 6: **Table S1.** Primer sequences used for gene expression analyses. (DOCX 14 kb)

## Abbreviations

AVPV: Anteroventral periventricular nucleus
Bdkrb2: B2 bradykinin receptor isoform XI
byr3: Byr3 zinc finger protein
Cbln4: Cerebellin 4 precursor gene
Cbx2: Chromobox protein homolog 2
Col3a1: Collagen type III alpha 1 chain
CPU: Central processing unit
Csrp3: Cysteine and glycine rich protein 3
Cul1: Cullin 1
Cul3: Cullin 3
Cyp1b1: Cytochrome P450 1B1
Dcn: Decorin
dCt: Delta cycle threshold
DE: Differential expression
Dmrt1: Doublesex and mab-3 related transcription factor 1
E3: Ubiquitin ligases
Eif2s3y: Eukaryotic translation initiation factor 2 subunit 3, Y-linked
Er71: Ets variant 2, *Etv2*
FCG: Four core genotype
FDR: False discovery rate
Fle1: Flexuosa
Fmod: Fibromodulin
GABA: Gamma-aminobutyric acid
Gapdh: Glyceraldehyde-3-phosphate dehydrogenase
GH: Growth hormone
Hdac2: Histone deacetylase 2
her: Hermaphrodite
HP1: Heterochromatin protein 1
iPSC: Induced pluripotent stem cells
Kat6al: Lysine acetyltransferase 6A-like
Kdm5d: Lysine Demethylase 5D
KRAB: Krueppel-associated box
KRAB-O: Krueppel-associated box only
Lum: Lumican precursor
Mb: Myoglobin
Mbd4: Methyl-CpG-binding domain protein 4
Mfn1: Mitofusin 1
Mfn2: Mitofusin 2
Mgrn1: Mahogunin ring finger 1
Mki67: Antigen KI-67 isoform X3
MPOA: Medial preoptic area
Myl1: Myosin light chain 1
Myl2: Myosin regulatory light chain 2
Nt: Nucleotides
Nt3: Neurotrophin 3
Ogn: Osteoglycin
Omd: Osteomodulin
PCA: Principal component analyses
Pja2: Praja ring finger ubiquitin ligase 2
PR: Progesterone receptor
QC: Quality-controlled
Rag2: V(D)J recombination-activating protein 2
RAM: Random-access memory
Rnf220: Ring finger protein 220
Rnf25: Ring finger protein 25
RTA: Reference transcriptome assembly
Sap30l: Sin3-Associated Protein P30-Like
Serpina3n: Serine (or cysteine) peptidase inhibitor, clade A, member 3 N
Setmar: SET domain and mariner transposase fusion gene
Shprh: SNF2 histone linker PHD RING helicase
Siah2: Siah E3 Ubiquitin Protein Ligase 2
Smpx: Small muscle protein X-linked
Sox9: Sry-related box 9
Spata7: Spermatogenesis Associated 7
SRY: Sex-determining region Y
Svs5: Seminal vesicle secretory protein 5
Tcf21: Transcription factor 21
TDF: Testis determining factor
Tex9: Testis-expressed protein 9
Trdn: Triadin
Zbtb44: Zinc finger and BTB domain-containing protein 44
Zfp112: Zinc finger protein 112
Zfp35: Zinc finger protein 35
Zfp410: Zinc finger protein 410
Zfp64: Zinc finger protein 64
Zfp655: Zinc finger protein 655
Zfp708: Zinc finger protein 708
Zfx: Zinc finger protein X-linked
Zfy: Zinc finger protein Y-linked
Zpr1: Zinc finger protein
