# Supplementary material for: Sexual dimorphism in brain transcriptomes of Amami spiny rats (Tokudaia osimensis): a rodent species where males lack the Y chromosome
Source: BMC Genomics. 2019 Jan 25;20:87. doi: 10.1186/s12864-019-5426-6 (PMC6347839; doi:10.1186/s12864-019-5426-6)
Supplement: Supplementary file 6 — Table S1. Primer sequences used for gene expression analyses. (DOCX 14 kb) [file 12864_2019_5426_MOESM6_ESM.docx]

Additional file 6: **Table S1** Primer sequences used for gene expression analyses

| Gene | **Direction** | **Primer Sequence (5’ 🡪 3’)** | **Amplicon Size** |
| --- | --- | --- | --- |
| *Gapdh* | Forward | GCCTCGTCCTGTAGACAAAA | 213 bp |
|  | Reverse | CAATTCTGAGCCTTGACTGT |  |
| *Svs5* | Forward | CCACTGATCTGGCTCTTGAT | 235 bp |
|  | Reverse | TTCGAGGAATACAGCCAAAG |  |
| *Cyp1b1* | Forward | GTGGCAATTCAAATGTCTCC | 163 bp |
|  | Reverse | TCACATTCAAGGGGTTCTGT |  |
| *Serpina* | Forward | ACCCTGAACATCAGGAGTCA | 189 bp |
|  | Reverse | TGTCCCATTGTCTTGGTCTT |  |
